# Who is the missing “matchmaker” between proteins and nucleic acids?

**DOI:** 10.1016/j.xinn.2021.100120

**Published:** 2021-05-21

**Authors:** Ping Xie

**Affiliations:** 1 Donghu Experimental Station of Lake Ecosystems, State Key Laboratory of Freshwater Ecology and Biotechnology, Institute of Hydrobiology, Chinese Academy of Sciences, Wuhan 430072, P.R. China; 2 Institute for Ecological Research and Pollution Control of Plateau Lakes, School of Ecology and Environmental Science, Yunnan University, Kunming 650500, P. R. China

## Main Text

Sixty years has passed since the cracking of the genetic code by Nirenberg and Matthaei in 1961; however, the origin of the code remains one of the greatest mysteries. It is great for a cell to innovate protein synthesis manipulated by RNA, and vice versa, which is a still unsolved “chicken-and-egg” paradox. Finding a solution is of crucial importance. Yet, most biologists hold a pessimistic view that no one knows exactly how the genetic code came into being, as an exact reconstruction of the process of the construction of genetic code may never be possible, or as there is no evidence in physics or chemistry of the control of chemical reactions by a sequence of any sort or of a code between sequences, and many papers have been published with titles indicating that their subject is the origin of the genetic code, while the content actually deals only with the evolution of the genetic code.^1^

## Deficiencies of previous theories

Currently, there are several prevalent hypotheses. The frozen accident hypothesis states that the allocation of codons to amino acids in a single ancestor occurred entirely by “chance” and then remained unchanged.^2^ The stereochemical hypothesis claims that there is, in many cases, a specific stereochemical fit between an amino acid and the base sequence of the corresponding codon on the appropriate tRNA.^3^ It is argued that the code is neither an accident nor totally frozen. The biosynthetic hypothesis postulates that the code is assigned in parallel to the evolution of amino acid biosynthesis.^4^ The genetic code is a product of selection, history, and chemistry.^5^ Since then, very little definitive progress has been made, although the literature is replete with attempts to explain the variation of the codes and possible rules of codon allocation to amino acids.

Unfortunately, all previous hypotheses have overlooked the importance of the energetic driving force, and none of them can explain the origination of the genetic code in the context of the biochemical system (a relation of part to whole) within a confined environment. They also ignored the relation between energy transformation and informatization (process of creating information). It is impossible to understand the origin of codons based on the codons alone or even by extending the perspective to the possible relationship between codons and amino acids.

## The ATP matchmaking hypothesis

To craft an objective self-contained model, I propose the logical argument that **only if “A” can handle two jobs (“B”, “C”) can a persistent (then heritable) connection between “B” and “C” be established (i.e., quasi-information) under selection**. Here, “B” and “C” are polynucleotides and polypeptides, respectively, whereas “A” is hidden. Previous attention was given neither to such logical reasoning nor to the matchmaker “A.” The phenomenal chicken-and-egg relation alone cannot give us the right answer.

**Figure 1:**
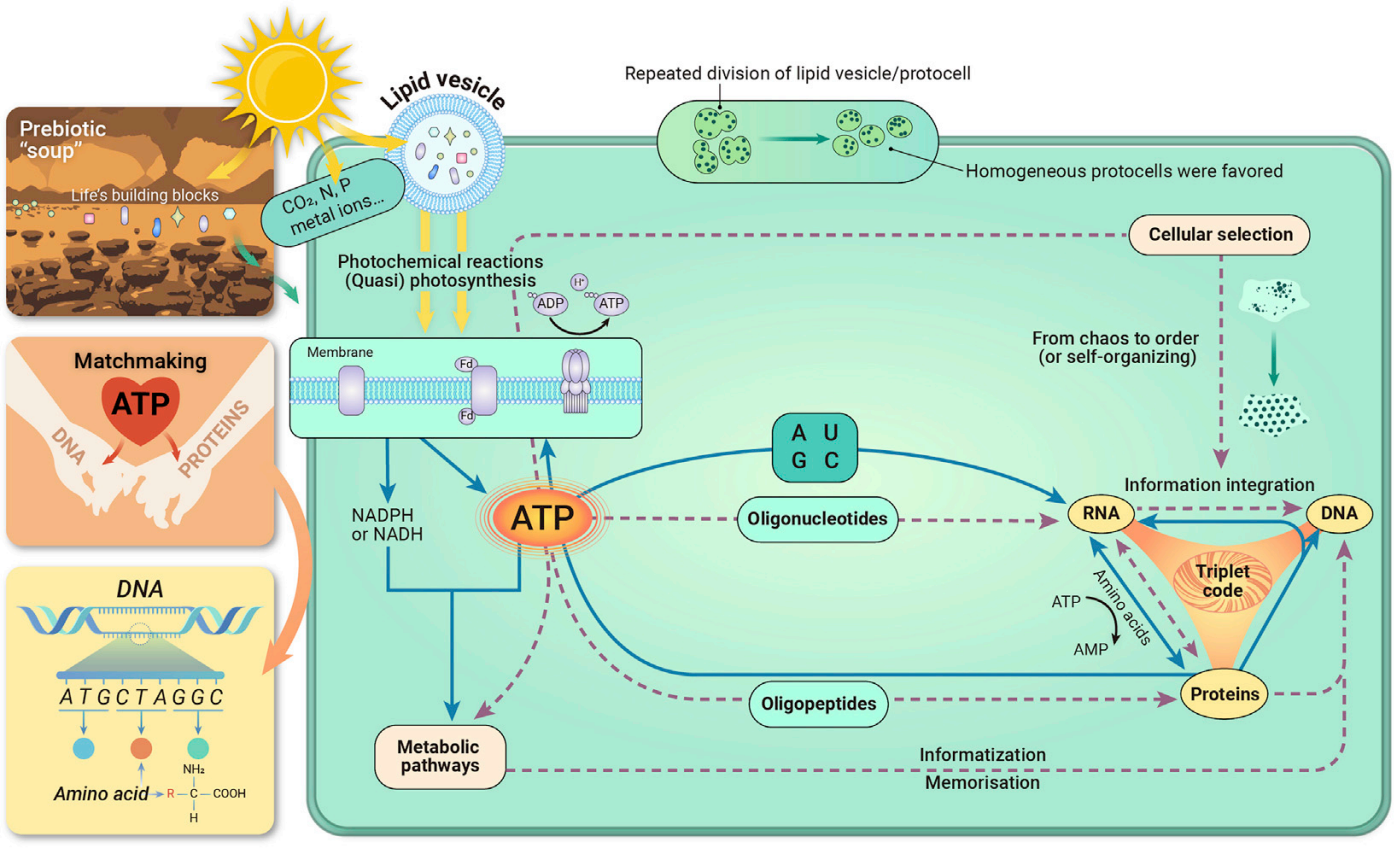
A new conceptual logic model showing how ATP energetically drove the origins of the genetic code and biochemical systems in the protocell based on the ATP matchmaking hypothesis Dashed red lines indicate protocellular processes, solid blue lines denote current cellular processes (not all of them are shown); and arrows indicate the direction of influences or actions.

Here, a new hypothesis is presented to explain how ATP acted as a “matchmaker” between biopolymers (although their honeymoon period has long been over), leading to the emergence of the genetic code (Figure 1). **ATP could fuel the elongation of both polynucleotides and polypeptides without additional energy input, making it possible to establish or fix molecular relationships between strings of nucleotides in polynucleotides and amino acids in polypeptides from their numerous random combinations throughout selection for specific sequences required for cellular survival.** To produce ATP efficiently, the protocell integrated various components and interactions into a sophisticated reaction system, that is, **with the development of periodic division, the protocell established a set of “intrinsic” attributes such as regularity, reproducibility, and rhythmicity of interactions, subsequently innovating biochemical pathways/cycles** as needed for survival or self-copying, and ultimately forming a complex molecular network. Meanwhile macromolecules became functionally differentiated, that is, some nucleotides carried amino acids (precursor of tRNA), and others built a platform for the synthesis of polypeptides (precursor of rRNA), which eventually replaced the irregular stochastic formation of polypeptides from amino acids activated by ATP. Polypeptides helped match the acceptor stem of tRNA to its anticodon, and the system developed the rule of codon-anticodon base pairing, that is, molecular recognition through stereochemical interactions (e.g., hydrogen bonds, van der Waals forces, and aromatic stacking), and ushered in a unified platform, rRNA, for protein synthesis according to a mRNA template. The polypeptides in turn participated not only in the construction of the transmembrane channels of hydrophilic molecules/ions but also in catalyzing the self-condensation of nucleotides. It is an astonishing imprint of the evolutionary route from RNA to DNA that RNA is still involved as a primer in the DNA replication of extant organisms. Subdivision of the genetic system (by subtle structural differences) into RNA and DNA might have favored orderly management and control of information in the very small cell in which hundreds of biochemical reactions occurred simultaneously. A triplet codon was completed with **cyclizing of polynucleotides and polypeptides into a feedback loop of reciprocal causation**. Consequently, the biochemical system achieved a status in which mRNA was immediately destroyed after the task was completed, while the genetic information recorded by DNA began to be permanently preserved and transmitted to ensure the continuation of a species. Then the Earth magically transitioned from chaotic prebiotic chemistry to attractive self-serving biology. It was this self-servingness that ignited exponential growth of biomolecules by incessant copying of homogeneous individuals. In short, ATP fueled the informatization of polynucleotides and polypeptides into heritable and functional molecules, although ATP was not the only molecule that could do this, but was favored by protocellular selection.

## Why ATP?

We can derive inspiration from the extant organization of biochemical systems to trace back to the origin of the genetic code. The birth of the genetic code must have been driven by some forces. Randomness and selection were frequently considered to be these forces, but I contend the energetic nature, as organisms are molecular machines in which numerous biochemical reactions take place for the acquisition, conversion, and use of energy.

ATP plays a central role in the energetics of biochemical systems across all extant and perhaps extinct life forms. It provides energy for metabolism through conversion of ATP/ADP/AMP, supporting the transformation of various biomolecules into each other in an exquisitely organized cell. The major metabolic pathways (e.g., the Calvin cycle, Embden-Meyerhof-Parnas pathway, and tricarboxylic cycle) are all coupled with ATP. The other four nucleotides for genetic coding are all derived from ATP and simpler life’s building blocks (LBB), as ATP provides biochemical force to these transformations. Therefore, ATP would be the most logical starting point for the origin of informatization.

## How ATP?

Accidental encapsulation does not make a cell, and the earliest cellular life must have resulted from long sequential processes with a plethora of intermediate forms. The present biosynthesis of ATP requires a transmembrane gradient of protons, making it exceedingly unlikely for macromolecules to evolve *in vitro,* as suggested by Eigen. On early Earth, fatty acids could automatically form lipid vesicles, permitting the passage of small molecules by diffusion such as CO_2_ and O_2_, but also functioning as a barrier for certain molecules and ions (e.g., H^+^), leading to different concentrations on the two sides of the membrane. Such an electro-chemical gradient could initiate energy management through polypeptide channels in the protocell.

The genetic code was likely innovated through processes from energy transformation to informatization, from ATP to triplet codon, and from RNA to DNA. The scenarios started from lipid vesicles enclosing sufficient LBB. Energy drove dazzling flows of their electrons and H^+^, causing prebiotic organosynthesis upon reaching the concentrations or energy thresholds required for polymerization. With the accumulation of large molecules and the perpetual input of small molecules, protocells likely reciprocated between cycles of enlargement and rupture, giving rise to periodic cell division.

Occasional polymerization and self-replication of molecules occurred on early Earth, but only fluent production of ATP facilitated by coevolution of macromolecules could establish cellular memorization, informatization and structuralization/compartmentalization. ATP eventually became the energetic core of a highly dynamic system through mediating and interlocking biochemical innovations. It is only the evolutionary completions of polynucleotide and polypeptide cyclizing into a feedback loop of reciprocal causation and preservation of the genetic code from RNA to DNA that, contrary to the central dogma, marked the dawn of cellular life or the last universal common ancestor (LUCA), when Darwinian evolution began to operate.

## Where to go?

Admittedly, all hypotheses explaining the origin of the genetic code or life are speculative, as billions of years have passed since life first appeared on Earth. It is, indeed, difficult to obtain an experimentally testable model from these hypotheses, since none of them can be verified or falsified through current methods. This pervasive speculation has been the case so far and may continue for the foreseeable future. In particular, the long parallel biochemical evolution in primordial cells could be an important reason for this difficulty.

In my view, a reliable hypothesis depends on its broad applicability in the interpretation of biochemical systems, as LUCA might have changed beyond recognition due to highly variable prokaryotic world. In contrast with previous hypotheses, the ATP matchmaking hypothesis provides a new logical framework, focusing on energetic force, coevolution of the genetic code with biochemical systems, and biological innovation from energy transformation to informatization. This hypothesis offers a more synthetic and mechanistic explanation than the existing dogmas that reflect separate, discrete aspects of the story. Moreover, it sheds light on the possible origins of cell division and biochemical systems, which have remained largely unknown thus far. As these are closely linked problems, understanding one requires understanding the other. Therefore, efforts to integrate multidisciplinary evidence and approaches are required to fully investigate how these biotic innovations were synergistically accomplished, with focus on energy transformation, so that we can reconstruct sequential, stepwise hierarchies of the genetic code. The present theory, by discovering the missing “matchmaker,” can yield a very plausible answer to the “DNA-Protein Paradox” that currently presents an impassable barrier to the naturalistic origin of life.
